# Genomic and biological characterization of three lytic phages targeting *Vibrio parahaemolyticus* and their application in a raw salmon model

**DOI:** 10.1128/spectrum.02617-26

**Published:** 2026-09-22

**Authors:** Miaosen Zhang, Xuejin Feng, Puyin Liu, Wu Qu, Min Jin

**Affiliations:** 1 State Key Laboratory Breeding Base of Marine Genetic Resource, Third Institute of Oceanography, Ministry of Natural Resources 118477, Xiamen, China; 2 Marine Science and Technology School, Zhejiang Ocean University 71233, Zhoushan, China; 3 College of Marine Sciences, Fujian Agriculture and Forestry University 12449, Fuzhou, China; 4 Southern Marine Science and Engineering Guangdong Laboratory (Zhuhai), Zhuhai, China; University of Minnesota Twin Cities, St. Paul, Minnesota, USA

**Keywords:** *Vibrio parahaemolyticus*, bacteriophage, queuosine modification, salmon, seafood safety

## Abstract

*Vibrio parahaemolyticus* is a major seafood-borne pathogen that poses substantial threats to food safety and aquaculture. However, effective and food-compatible strategies for controlling this pathogen along the seafood supply chain remain limited. In this study, three novel lytic bacteriophages (Z5, Z44, and Z109) that infect *V. parahaemolyticus* were isolated from seafood-market wastewater and characterized with respect to their biological properties, genomic features, and bactericidal activity under food-relevant conditions. All three phages exhibited siphovirus-like morphologies and short latent periods (~10 min), with Z5 and Z44 showing high burst sizes (267–688 plaque-forming units [PFU]/cell). The three phages showed stability across a wide range of temperatures (4°C–60°C) and pH values (4–10), supporting their compatibility with seafood processing and cold-chain storage. Phylogenetic analyses indicated that Z5 and Z44 represent novel species within the family *Queuovirinae*, whereas Z109 belongs to the genus *Mardecavirus*. Genomic analyses confirmed the absence of known antibiotic resistance genes or virulence factors, supporting their genetic safety for food applications. Predicted interactions between phage- and host-encoded anti-restriction and defense systems likely influence phage lytic efficacy and killing patterns. Specifically, Z5 and Z44 encode a putative 7-deazaguanine DNA modification pathway that may enhance resistance to host restriction-modification systems. Conversely, the presence of prophage-related regions in the host genome may contribute to the reduced killing efficacy of Z109 at high phage doses. In a salmon model, all three phages significantly reduced *V. parahaemolyticus* at 25°C and 4°C, demonstrating effective biocontrol in a food matrix. In addition, the phages strongly inhibited biofilm formation, although their capacity to disrupt mature biofilms was limited. Collectively, these results indicate that Z5, Z44, and Z109 are promising candidates for the biocontrol of *V. parahaemolyticus* contamination in seafood systems.

**IMPORTANCE:**

Bacterial infections in aquaculture and seafood pose significant challenges to food safety, with *Vibrio parahaemolyticus* being a major seafood-borne pathogen. Bacteriophages are promising alternatives to antibiotics, but their effectiveness may be influenced by phage-encoded counter-defense strategies and host-encoded antiphage defense systems. In this study, we characterized three novel lytic phages and observed distinct killing patterns under different infection conditions. In a raw salmon model, these phages effectively reduced *V. parahaemolyticus*, demonstrating their practical biocontrol potential. Genome analysis identified putative phage-encoded DNA modification genes and host prophage-associated defense genes that may help explain these differences. These findings highlight the importance of considering both phage and host genomic features when optimizing phage-based strategies for controlling *V. parahaemolyticus* in aquaculture and seafood systems.

## INTRODUCTION

*Vibrio parahaemolyticus* is a gram-negative halophilic bacterium widely distributed in marine environments and represents a major cause of seafood-associated foodborne illness (1). It also causes important diseases in aquaculture, including acute hepatopancreatic necrosis disease and highly lethal *Vibrio* disease (HLVD) in shrimp, resulting in substantial economic losses to the aquaculture industry (2). These aquaculture-associated infections further increase the risk of pathogen entry into the seafood supply chain. In China, *V. parahaemolyticus* has been identified as one of the leading bacterial causes of food poisoning, accounting for 9.6% of cases in Shandong (2011–2016) and 33.5% in Shanghai (2010–2020) (3, 4). In the United States, approximately 35,000 cases of foodborne illness per year are estimated to result from *V. parahaemolyticus* (5). Human infection with *V. parahaemolyticus* most commonly results in acute gastroenteritis, and severe cases may progress to septicemia, particularly in individuals with underlying liver disease (6).

The primary route of *Vibrio* infection is the consumption of contaminated raw or undercooked seafood (7). Aquatic products, especially fish consumed raw, such as salmon, are susceptible to microbial contamination at multiple stages of the seafood supply chain (8). Traditional control strategies in the seafood industry include chemical sanitization, thermal processing, and modified-atmosphere packaging. Although effective in reducing bacterial loads, these approaches may be constrained by concerns related to antimicrobial resistance, adverse effects on sensory quality, and incompatibility with cold-chain storage conditions (9). Consequently, alternative antimicrobial strategies, including bacteriophages and phage-derived enzymes, are increasingly being explored for the control of *V. parahaemolyticus* in seafood production and processing environments (10).

Bacteriophages are natural predators of bacteria and have been extensively investigated as biocontrol agents against bacterial pathogens. Compared with conventional sterilization methods, phages can selectively reduce bacterial contamination during food production and processing while preserving sensory quality (11). Their self-amplifying nature enables replication specifically in the presence of susceptible bacterial hosts, thereby enhancing antibacterial efficacy. In the absence of hosts, phage particles are readily degraded, minimizing residual accumulation in food matrices compared with persistent chemical sanitizers. However, the host specificity and narrow host range of phages may limit their effectiveness against genetically diverse pathogen populations, underscoring the importance of isolating and composing multiple efficient lytic phages for reliable biocontrol applications.

Phages intended for food use should be strictly lytic, free of known antibiotic resistance and virulence genes, and stable across a range of pH values and low to moderate temperatures typically encountered in food storage and handling conditions. Consumer demand for chemical-free and pathogen-free foods has driven the development of bacteriophage-based antimicrobial products, which are currently applied mainly against *Listeria monocytogenes*, *Salmonella*, and *Escherichia coli*. Several studies have demonstrated that bacteriophages can effectively reduce *L. monocytogenes* contamination in ready-to-eat foods and food processing environments (12, 13). Similarly, bacteriophages targeting Salmonella have been widely investigated and shown to significantly reduce bacterial loads in various food matrices, including poultry products and fresh produce (14). However, seafood products with high fat and protein contents may interfere with phage adsorption, diffusion, and host-phage interactions, thereby reducing antimicrobial efficacy in real food systems (15). In particular, fatty fish products, such as salmon, may require higher multiplicities of infection (MOI) or alternative application strategies to achieve effective bacterial reduction, highlighting the need for matrix-specific optimization in seafood biocontrol. This aspect remains underexplored in *V. parahaemolyticus* phage studies. These matrix-associated constraints further emphasize the importance of identifying phage candidates with high infection efficiency and strong bactericidal activity. Therefore, the targeted discovery and characterization of high-performance *V. parahaemolyticus* phages remain important challenges.

In the present study, three novel bacteriophages (Z5, Z44, and Z109) targeting *V. parahaemolyticus* were isolated and systematically characterized based on morphological features, genomic properties, biological characteristics, and antibacterial performance. Their efficacy was further evaluated using a raw salmon model and antibiofilm assays to assess their potential applications in seafood preservation. Beyond conventional phage characterization, this study integrated comparative genomic analysis with biological phenotyping to explore potential genetic factors associated with differences in phage infection performance. Notably, our finding of an unusual MOI-dependent killing pattern and potential host-associated defense elements provided additional perspectives for understanding phage-host interactions and optimizing phage-based biocontrol strategies.

## MATERIALS AND METHODS

### Bacterial strains and culture conditions

Two *V. parahaemolyticus* strains, vp-201911 and vp-HL-202005, were used as host bacteria for phage isolation. These pathogenic strains were originally isolated from *Penaeus vannamei* post-larvae in diseased ponds (16). Strain vp-HL-202005 has been identified as a highly lethal *V. parahaemolyticus* strain and is associated with HLVD, whereas vp-201911 is regarded as a conditionally pathogenic strain under experimental infection conditions (16). All *Vibrio* strains used in this study were cultured in 2216E liquid medium (12.5 mM tryptone, 5 mM yeast extract, and artificial seawater, pH 7.5) at 30°C with shaking at 180 rpm. For solid cultivation, *Vibrio* strains were plated on either 2216E agar (1.5% agar) or thiosulfate citrate bile salt sucrose agar (TCBS; Haibo Microbial, China), and incubated at 30°C unless otherwise stated.

### Phage isolation

Phages were isolated from wastewater samples collected at seafood markets in Xiamen City, Fujian Province, and Zhoushan City, Zhejiang Province, using *V. parahaemolyticus* as the host, based on a previously described method with minor modifications (17). Briefly, equal volumes of 0.45 μm filtered wastewater were mixed with early log-phase cultures of *V. parahaemolyticus* (OD_600_ = 0.3) and incubated at 30°C under shaking at 180 rpm for 8 h to enrich *V. parahaemolyticus*-infecting phages. Following incubation, cultures were filtered through a 0.45 μm membrane to remove bacterial cells, and the resulting filtrates were screened for plaques using the double-layer agar method. For plaque screening, filtrates were serially diluted, and 1 mL of each dilution was mixed with 1 mL of log-phase *V. parahaemolyticus* (OD_600_ = 0.3), followed by incubation at room temperature for 10 min to allow phage adsorption. The mixture was then added to 4 mL of 2216E soft agar (0.5%, ~50°C) and poured onto 2216E agar (1.5%) plates to form a double-layer agar. After incubation at 30°C, individual plaques were picked and purified through five successive rounds of double-layer agar plating. The final purified plaques were suspended in SM buffer (0.01% [wt/vol] gelatin, 8 mM MgSO_4_, 50 mM Tris-HCl, 100 mM NaCl, pH 7.5) at 4°C.

### Phage amplification and purification

For phage amplification, 1 mL of phage stock was added to 30 mL of early-log phase host culture (OD_600_ = 0.3) and incubated at 30°C with shaking at 180 rpm until bacterial lysis was observed. The lysate was centrifuged at 8,000 *× g* for 10 min at 4°C, and the supernatant was filtered through a 0.45 μm membrane to obtain the phage suspension. The resulting suspension was treated with DNase I and RNase A (final concentration, 1 μg/mL each) at 4°C for 1 h to degrade residual host nucleic acids. NaCl was then added to a final concentration of 1 M, followed by incubation at 4°C for 1 h to promote phage precipitation. Polyethylene glycol 8000 (PEG 8000) was added to a final concentration of 10% (wt/vol), and the mixture was incubated at 4°C for 1–3 days to precipitate phage particles. Precipitated phages were collected by centrifugation at 12,000 *× g* for 60 min at 4°C and resuspended in SM buffer. For further purification, the phage suspension was subjected to cesium chloride density gradient ultracentrifugation at 200,000 *× g* and 4°C for 24 h. The visible phage bands were collected using a sterile syringe and dialyzed against sterile SM buffer to remove cesium chloride. Final phage preparations were stored at 4°C in the dark for subsequent experiments.

### Transmission electron microscopy

Phage morphology was examined by transmission electron microscopy (TEM) using negative staining with 1% phosphotungstic acid (18). Briefly, 10 μL of phage concentrate was applied to 200-mesh copper grids and incubated in the dark for 10–30 min. The grids were then stained with 1% phosphotungstic acid for 20 min, air-dried for 30 min, and observed under a TEM (Hitachi HT-7800, Japan) at 80 kV. Phage particle dimensions were measured from TEM images using ImageJ 1.8.0 (*n* = 5).

### Biological characterization of phages

One-step growth curves of phages were determined following a previously reported method with minor modifications (19). *V. parahaemolyticus* and phage suspensions were mixed at an MOI of 0.001 and incubated in the dark for 5–10 min to allow adsorption, depending on the adsorption characteristics of each phage. The mixture was centrifuged at 8,000 *× g* and 4°C for 5–10 min, and the pellet was resuspended in 1 mL of 2216E medium. This centrifugation and resuspension step was repeated twice to remove unbound phages. The final suspension was added to fresh 2216E medium and incubated at 30°C under shaking at 180 rpm. Samples were collected every 5–10 min over a 1 h period, and phage titers were determined using the double-layer agar plating method. Time zero was defined as the transfer of the washed, resuspended bacteria-phage mixture into fresh medium. The latent period was determined from the time point after removal of unadsorbed phages and initiation of synchronized infection. The burst size was calculated as the ratio of phage particles released at the plateau phase to the number of initially infected cells after removal of unadsorbed phages.

Temperature, pH, and chloroform stability of the phages were assessed according to Cao et al. (20) with minor modifications. For thermal stability, 1 mL of phage suspension in SM buffer (pH 7.5) was incubated at 4°C, 10°C, 20°C, 30°C, 40°C, 50°C, 60°C, and 70°C for 1 h. For pH stability assays, 100 μL of phage suspension was mixed with 0.9 mL of phosphate-buffered saline and incubated at pH 4.0, 6.0, 8.0, 10.0, and 12.0 at 30°C for 1 h. For chloroform sensitivity assays, 1 mL of phage suspension was incubated with 50 μL of chloroform or sterile water at 30°C for 1 h. Residual phage titers were determined using the double-layer agar plating method. All experiments were conducted in triplicate with independent biological replicates (*n* = 3).

### Determination of host range

Host range was evaluated by spot assay conducted to assess the host range of purified *Vibrio* phages (21). Early log-phase *Vibrio* cultures (2 mL; OD_600_ = 0.3) were mixed with 4 mL of molten 2216E soft agar and overlaid onto 2216E agar plates (1.5% agar). After solidification, 5 μL of phage suspension, approximately ~10⁸ plaque-forming units (PFU)/mL, was spotted onto the bacterial lawn, and then incubated at 30°C. In addition, potential host associations were predicted *in silico* using iPHoP (22), and only predictions with retention probability scores greater than 90% were retained for further analysis. These predictions are merely preliminary calculations intended to indicate the potential host range and require further experimental verification.

### Phage genomic DNA extraction

Phage genomic DNA was extracted using the phenol–chloroform method (23). The phage concentrate was treated with proteinase K (0.2 mg/mL), sodium dodecyl sulfate (10%, wt/vol), and ethylenediaminetetraacetic acid (0.5 mol/L, pH 8.0), followed by incubation at 65°C for 3 h. After enzymatic digestion, an equal volume of phenol–chloroform–isoamyl alcohol (25:24:1, vol/vol/vol) was added, mixed thoroughly, and centrifuged at 12,000 *× g* for 5 min at 4°C. The aqueous phase was carefully transferred to a fresh tube, and extraction with phenol–chloroform–isoamyl alcohol was repeated twice. The recovered aqueous phase was mixed with an equal volume of chloroform–isoamyl alcohol (24:1, vol/vol), vigorously shaken for 30 s, and centrifuged at 12,000 *× g* for 5 min at 4°C. The resulting aqueous phase was combined with an equal volume of isopropanol and incubated at −20°C overnight. After centrifugation at 12,000 *× g* for 5 min at 4°C, the DNA pellet was washed twice with pre-chilled 70% ethanol, air-dried, dissolved in 100 μL of Tris-EDTA buffer (10 mM Tris-HCl, 1 mM EDTA, pH 8.0), and stored at −80°C.

### Phage-genome sequencing and analysis

Complete phage genomes were sequenced by Ruixing Bio-Tech (Shanghai, China). Libraries were prepared using the NEBNext Ultra DNA Library Prep Kit (NEB, USA) with an average insert size of approximately 300 bp, and sequencing was performed on the Illumina Nova platform using 2 × 150 bp paired-end reads. Raw data were processed with Trimmomatic (v.0.32) and assembled using Velvet (v.1.2.03) (24, 25). Open reading frames (ORFs) were predicted using GeneMarkS v.4.28 (26) and Glimmer3 v.3.02 (27). Functional annotation was performed by BLASTP searches against the NCBI non-redundant protein database (E-value < 10⁻⁵), and viral protein classification was conducted by comparison with the Prokaryotic Virus Remote Homologous Groups database (28). Transfer RNAs (tRNAs) were detected with tRNAscan-SE (v2.0) (29). Antibiotic resistance genes were screened using the Comprehensive Antibiotic Resistance Database (30), and virulence genes were detected using the Virulence Factor Database (31). Genome visualization was performed using Proksee (27). Phage termini and DNA packaging strategies were inferred using PhageTerm (32). The complete genome sequences of phages Z5, Z44, and Z109 were deposited in the GenBank database under accession numbers PX979761, PX979762, and PX979763, respectively.

### Phylogenetic and taxonomic analyses

Taxonomic placement of the three phages was assessed using a protein-sharing network with vConTACT2 (v.0.11.3) based on the ProkaryoticViralRefSeq94-Merged database and visualized in Cytoscape (v.3.9.1) (33, 34). Phages exhibiting the highest nucleotide similarity to the three phages were identified by BLASTn analysis against the NCBI GenBank database, and the top 12 most closely related genomes were selected for downstream phylogenetic analyses. Whole-genome phylogenetic relationships were inferred using VICTOR with the Genome BLAST Distance Phylogeny (GBDP) approach and default parameters (35). Genome-wide similarity was calculated using the Virus Intergenomic Distance Calculator (VIRIDIC) under default settings (Moraru et al., 2020)(36). Taxonomic classification followed genome-wide similarity criteria established by the International Committee on Taxonomy of Viruses (ICTV) (37). Comparative genomic analysis of phage genomes was performed using Clinker (v.0.0.31) (38).

### Determination of *in vitro* phage bactericidal efficacy

*In vitro* bactericidal efficacy assays were conducted as previously described, with minor modifications (39). *V. parahaemolyticus* cultures were grown to logarithmic phase (OD_600_ = 0.3) in 2216E medium, aliquoted, and mixed with an equal volume of phage suspension at MOIs of 0.01, 1, or 100. The mixtures were incubated in 96-well plates in a Varioskan LUX multimode microplate reader (Thermo Fisher Scientific, USA) at 30°C under shaking at 180 rpm for 12 h. Bacterial growth was monitored by measuring OD_600_ at 30 min intervals. SM buffer and kanamycin (0.5 mg/mL) served as negative and positive controls, respectively. For each phage, the *in vitro* bactericidal efficacy was determined in three independent biological replicates.

### Determination of phage bactericidal efficacy in raw salmon model

Salmon fillets were obtained from a retail supermarket in Xiamen, China. Samples were aseptically cut into 1.5 cm × 1.5 cm × 1.5 cm cubes using a sterile scalpel and exposed to ultraviolet irradiation for 30 min to reduce indigenous surface microbiota. To simulate contamination, a *V. parahaemolyticus* strain suspension (10⁸ CFU/mL) was sprayed onto the surface of each salmon cube (approximately 100 μL per cube), followed by incubation at 30°C for 1 h to allow adhesion of bacteria. Phage suspensions were then applied to the contaminated cubes at MOI 100 and 1. Treated samples were stored at 4°C or at ambient temperature (24°C ± 1°C) to simulate practical application conditions. SM buffer-treated samples served as negative controls. Samples were collected at 2, 4, and 6 h after treatment, homogenized in 25 mL sterile PBS, and bacterial counts were determined by plating on TCBS agar. All treatments were conducted with three independent biological replicates.

### Biofilm prevention and disruption by phage activity

Biofilm prevention and disruption assays were performed using previously reported methods with minor modifications (40). For the biofilm prevention assay, phages Z5, Z44, and Z109 were individually mixed with *V. parahaemolyticus* at MOIs 1,000, 100, and 10, respectively. The mixtures were then added to 96-well microplates and incubated at 30°C for 12 h. After incubation, supernatants were discarded, and each well was gently washed three times with 200 μL PBS to remove non-adherent cells. The wells were then fixed with 200 μL methanol for 30 min, stained with 1% (wt/vol) crystal violet for 20 min, and washed three times with PBS. The bound crystal violet was solubilized using 33% (vol/vol) glacial acetic acid. Biofilm biomass was quantified by measuring OD_600_ using a microplate reader (Thermo Fisher Scientific, USA).

For the biofilm disruption assay, 200 μL of *V. parahaemolyticus* suspension (10^7^ CFU/mL) in 2216E liquid medium was added to 96-well microplates and incubated at 30°C for 48 h. The wells were then gently washed three times with PBS to remove non-adherent cells. Phage concentrates (10¹¹ PFU/mL) were added at an MOI of 1,000, followed by the addition of fresh 2216E liquid medium. Three replicate 96-well microplates were incubated at 30°C for 180 min. At 60, 120, and 180 min, one 96-well microplate was sampled at each time point, gently washed with PBS, and biofilm biomass was quantified by OD_600_ measurement as described above. All treatments were performed with three independent biological replicates.

### Statistical analysis

Statistical analyses were performed using GraphPad Prism (v.10). Data are reported as mean ± standard deviation (SD). For temperature and pH stability assays, differences among multiple treatment groups were evaluated using one-way analysis of variance (ANOVA), followed by Tukey’s post hoc multiple comparison test. For chloroform sensitivity assays, comparisons between two groups were analyzed using Student’s *t*-test. For biofilm prevention assays, one-way ANOVA, followed by Dunnett’s post hoc test, was applied. For biofilm disruption assays involving both treatment and incubation time, two-way ANOVA, followed by Tukey’s multiple comparisons test, was employed. For phage therapy experiments on raw salmon, one-way ANOVA, followed by Dunnett’s post hoc test, was employed. A *P* value < 0.05 was considered statistically significant.

## RESULTS

### Morphological characteristics of phage plaques and particles

**Fig 1:**
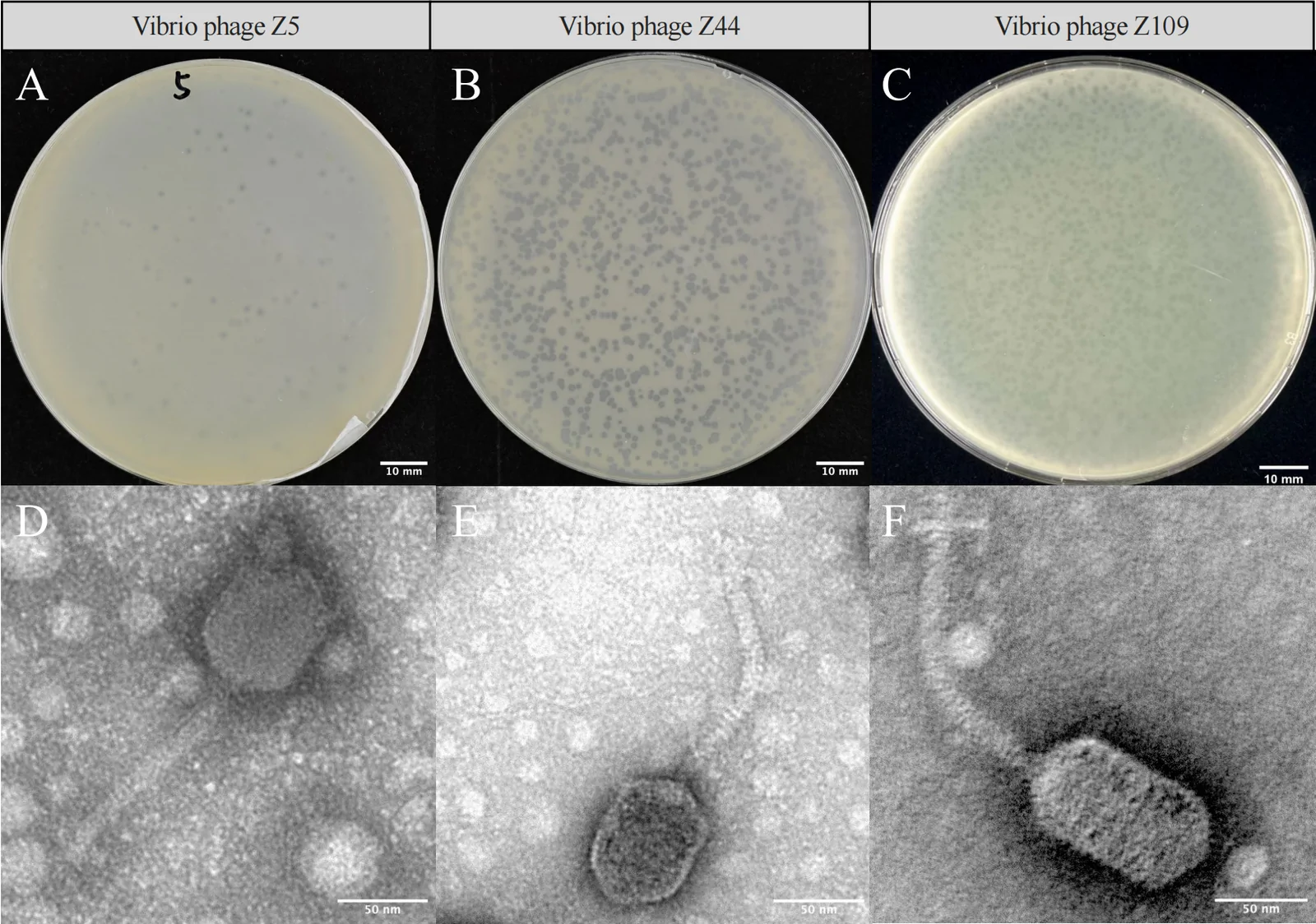
Morphological characteristics of phage plaques and particles. (**A–C**) Plaques formed by lytic phages Z5, Z44, and Z109 on their respective host bacterial lawns after 12 h of incubation (scale bar = 10 mm). (**D–F**) Transmission electron micrographs of phage Z5, Z44, and Z109 particles (scale bar = 50 nm).

Using *V. parahaemolyticus* vp-201911 as the host strain, two lytic phages, Z5 and Z44, were isolated from wastewater collected at seafood markets in Xiamen City, Fujian Province, China. A third lytic phage, Z109, was isolated from wastewater collected at a seafood market in Zhoushan City, Zhejiang Province, China, using *V. parahaemolyticus* vp-HL-202005 as the host strain. All three phages produced distinct, well-defined plaques on their respective bacterial lawns, with mean plaque diameters of 1.71 ± 0.43 mm (Fig. 1A), 1.60 ± 0.17 mm (Fig. 1B), and 1.30 ± 0.13 mm (Fig. 1C), respectively. Phage Z5 produced clear plaques surrounded by halos measuring 3.7 ± 0.44 mm in diameter. Phage Z44 formed plaques with clear centers, whereas Z109 generated less distinct, diffuse plaques.

Transmission electron microscopy indicated that all three phages exhibited siphovirus-like morphology characterized by long, non-contractile tails. Phage Z5 possessed an icosahedral head with a diameter of 74 ± 2 nm and a long, non-contractile tail measuring 121 ± 9 nm (Fig. 1D). Phage Z44 displayed a polyhedral head (74 ± 1 nm) and a long, non-contractile tail measuring 147 ± 4 nm (Fig. 1E). Phage Z109 exhibited an elongated head measuring 101 ± 2 nm and a long, non-contractile tail of 173 ± 7 nm (Fig. 1F).

### Biological characteristics of phages

**Fig 2:**
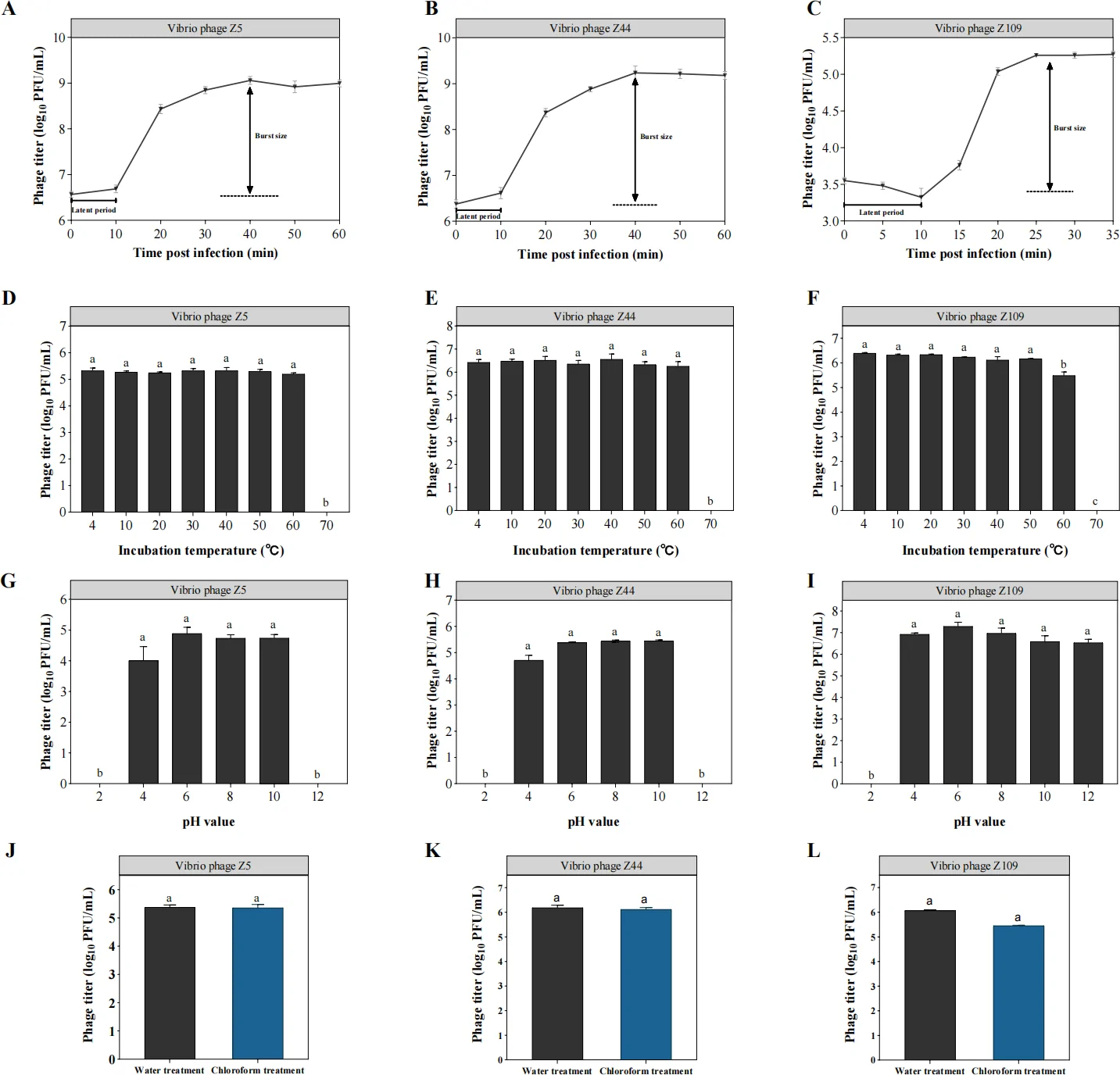
Biological characteristics of phages Z5, Z44, and Z109. (**A–C**) One-step growth curves of phages Z5, Z44, and Z109. (**D–F**) Thermal stability of phages Z5, Z44, and Z109 at different temperatures. (**G–I**) pH stability of phages Z5, Z44, and Z109. (**J–L**) Chloroform sensitivity of phages Z5, Z44, and Z109. For panels **D–I**, statistical differences among multiple groups were assessed using one-way ANOVA, followed by Tukey’s post hoc multiple comparison test. For panels **J–L**, statistical significance between two groups was evaluated using Student’s *t*-test. Different lowercase letters above the bars indicate statistically significant differences among groups (*P* < 0.05), whereas bars sharing the same letter are not significantly different. All data are presented as mean ± standard deviation (SD) from three independent biological replicates (*n* = 3).

The life cycle of the three lytic phages, Z5, Z44, and Z109, was determined using one-step growth curve analysis (Fig. 2A through C). All three phages exhibited a short latent period of approximately 10 min following infection, after which phage titers increased. Z5 and Z44 titers reached a plateau at approximately 30 min post-infection, whereas Z109 titers reached a plateau at approximately 20 min post-infection. The burst sizes of Z5, Z44, and Z109 were 267 ± 15 PFU/cell, 688 ± 50 PFU/cell, and 52 ± 1 PFU/cell, respectively.

Figures 2D through F depict the thermal stability of the three phages. Phage titers remained stable across a temperature range of 4°C–50°C. At 60°C, Z5 and Z44 remained relatively stable, whereas Z109 exhibited a significant reduction in titer of 79.6% compared with that at 50°C (*P* < 0.05). All three phages lost detectable activity at 70°C. Figures 2G through I show the pH stability of the three phages. Phage Z109 exhibited a relatively broad pH tolerance, maintaining stable titers between pH 4 and pH 12. In contrast, Z5 and Z44 remained stable within a narrower pH range (pH 4–10), and at pH <2 or >12, titers declined below the detection limit. Figures 2J through L illustrate the tolerance of the three phages to 5% chloroform. Exposure to 5% chloroform had no significant effect on phage titers, indicating that the particles lack chloroform-sensitive lipid components.

### Host range of phages

Spot assay results showed that two of eleven tested *Vibrio* strains were sensitive to Z109, namely *V. parahaemolyticus* vp-HL-202005 and *V. parahaemolyticus* FJ3 (Table 1). Host prediction using iPHoP, a tool with high reported recall and precision for phage-host association, identified 15 potential *Vibrio* hosts for Z109, encompassing at least 13 *Vibrio* species (Table S1), suggesting a potentially broader host range. However, these predictions require further experimental validation. In contrast, Z5 and Z44 exhibited a narrow host range, with no additional hosts detected by either spot assays or iPHoP prediction. This apparent specificity may reflect the limited number of *Vibrio* strains tested or intrinsic constraints in phage receptor recognition.

**TABLE 1:** Host range of phage Z5, Z44, and Z109 based on spot assays^*a*^

Species	Strain	Phage sensitivity		
		Z5	Z44	Z109
*V. parahaemolyticus*	vp-201911	+	+	−
*V. parahaemolyticus*	vp-HL-202005	−	−	+
*V. parahaemolyticus*	FJ3	−	−	+
*V. parahaemolyticus*	AP5	−	−	−
*V. parahaemolyticus*	AP7	−	−	−
*V. parahaemolyticus*	AP10	−	−	−
*V. parahaemolyticus*	AP11	−	−	−
*V. harveyi*	AP1	−	−	−
*V. harveyi*	AP2	−	−	−
*V. harveyi*	AP3	−	−	−
*V. campbellii*	AP8	−	−	−

^*a*^ “+” indicates that the Vibrio strain was susceptible to the corresponding phage, whereas “–” indicates that the Vibrio strain was not susceptible to the corresponding phage.

### Genomic characteristics and comparative genome analysis

**Fig 3:**
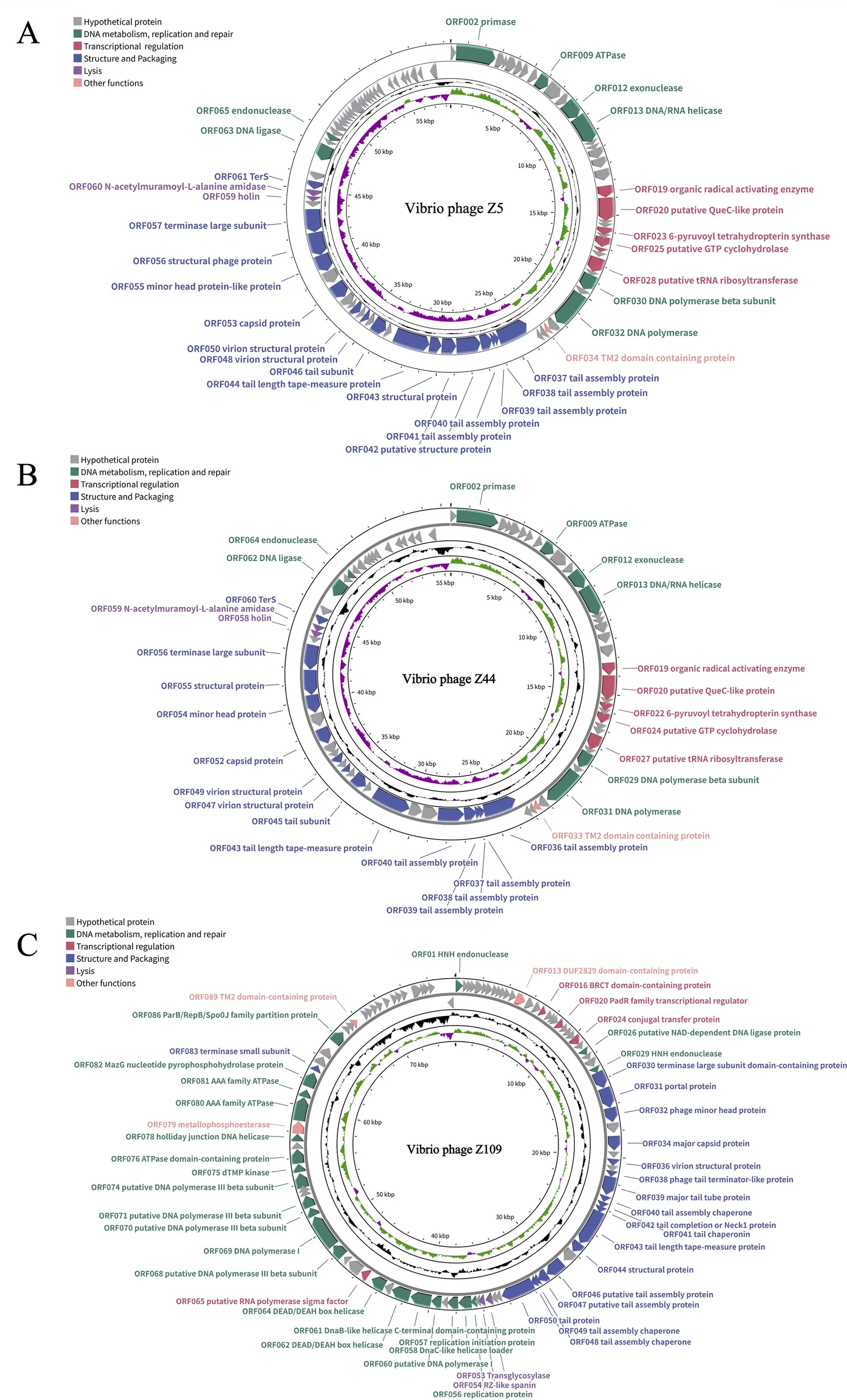
Genome maps of phages Z5 (**A**), Z44 (**B**), and Z109 (**C**). From outer to inner circles: (1) functionally annotated ORFs; arrow orientation indicates transcriptional direction; different colors indicate different functional modules; (2) GC content (%); (3) GC skew; and (4) genome coordinates.

The complete genomes of the three phages were sequenced and analyzed, and their main genomic features are summarized in Table S2. All three phages possess linear double-stranded DNA genomes, with sizes ranging from 55,517 to 76,073 bp and GC contents of 46.24%–48.89%. ORF prediction indicated that phages Z5, Z44, and Z109 encode 87 (Fig. 3A), 76 (Fig. 3B), and 102 (Fig. 3C) ORFs, respectively. Of these, 32, 30, and 49 ORFs were assigned putative functions, respectively. These genes are primarily associated with essential steps in the phage life cycle, including structural assembly, DNA metabolism, replication and repair, transcriptional regulation, and host lysis. Searches against the Comprehensive Antibiotic Resistance Database and the Virulence Factor Database detected no known antibiotic resistance or virulence-associated genes in any of the three genomes. A single tRNA gene was identified in the genomes of Z5 and Z44, whereas no tRNA genes were detected in the Z109 genome.

PhageTerm analysis indicated that Z5 and Z44 possess linear genomes with terminal redundancy and circular permutation, consistent with a headful (pac-type) DNA packaging mechanism. The genome assemblies were supported by high sequencing depth, with average coverage values of 101,884× (Z5), 83,700× (Z44), and 86,852× (Z109), respectively. All three genomes were assembled from Illumina data sets consisting of 21,314,187, 17,094,160, and 24,094,303 paired-end reads (Z5, Z44, and Z109, respectively). Since DNA is packaged to a fixed capsid capacity in headful (pac-type) phages, shorter genome length is recommended to reduce replication burden and increase virion yield per infection cycle (41, 42). Consistent with this interpretation, Z5, which has a slightly larger genome (59,127 bp), exhibited a lower burst size (267 ± 15 PFU/cell) than Z44 (56,216 bp, 688 ± 50 PFU/cell) when infecting the same host. Phage Z109 also appears to use a pac-type packaging strategy but is characterized by a single highly significant pac site located on the reverse strand at position 75,560 bp, together with multiple preferred termini on the forward strand, indicating terminal redundancy and partial circular permutation. Nearly all ORFs of Z109 are encoded on the plus strand, with only a single ORF on the minus strand containing the pac site. This arrangement may promote directional headful genome packaging while preserving uninterrupted transcription of continuous plus-strand ORFs. In contrast, Z5 and Z44 exhibited pronounced strand-specific functional organization. Plus-strand genes predominantly encode proteins involved in nucleic acid metabolism, DNA replication, and tRNA or nucleoside modification (e.g., DNA polymerases, exonucleases, helicases, and queuosine modification enzymes), whereas minus-strand genes mainly encode proteins associated with virion morphogenesis and host lysis, including tail assembly proteins, capsid proteins, terminases, holins, and endolysins. This strand-specific organization likely supports temporal regulation of the phage life cycle by separating early replication functions from late virion assembly and lysis functions, thereby improving propagation efficiency.

Comparative genomic analysis further showed that Z5 and Z44 share high sequence similarity (98.41% identity across 93% of their genomes), whereas no significant nucleotide-level similarity was detected between either of these phages and Z109. Despite this divergence, two tail-associated genes were highly conserved among all three phages (Fig. 5B). This pattern is consistent with the mosaic architecture typical of bacteriophage genomes (43) and with strong functional constraints on structural genes involved in tail assembly and host recognition (44).

### Phylogenetic and taxonomic classification of phages

**Fig 4:**
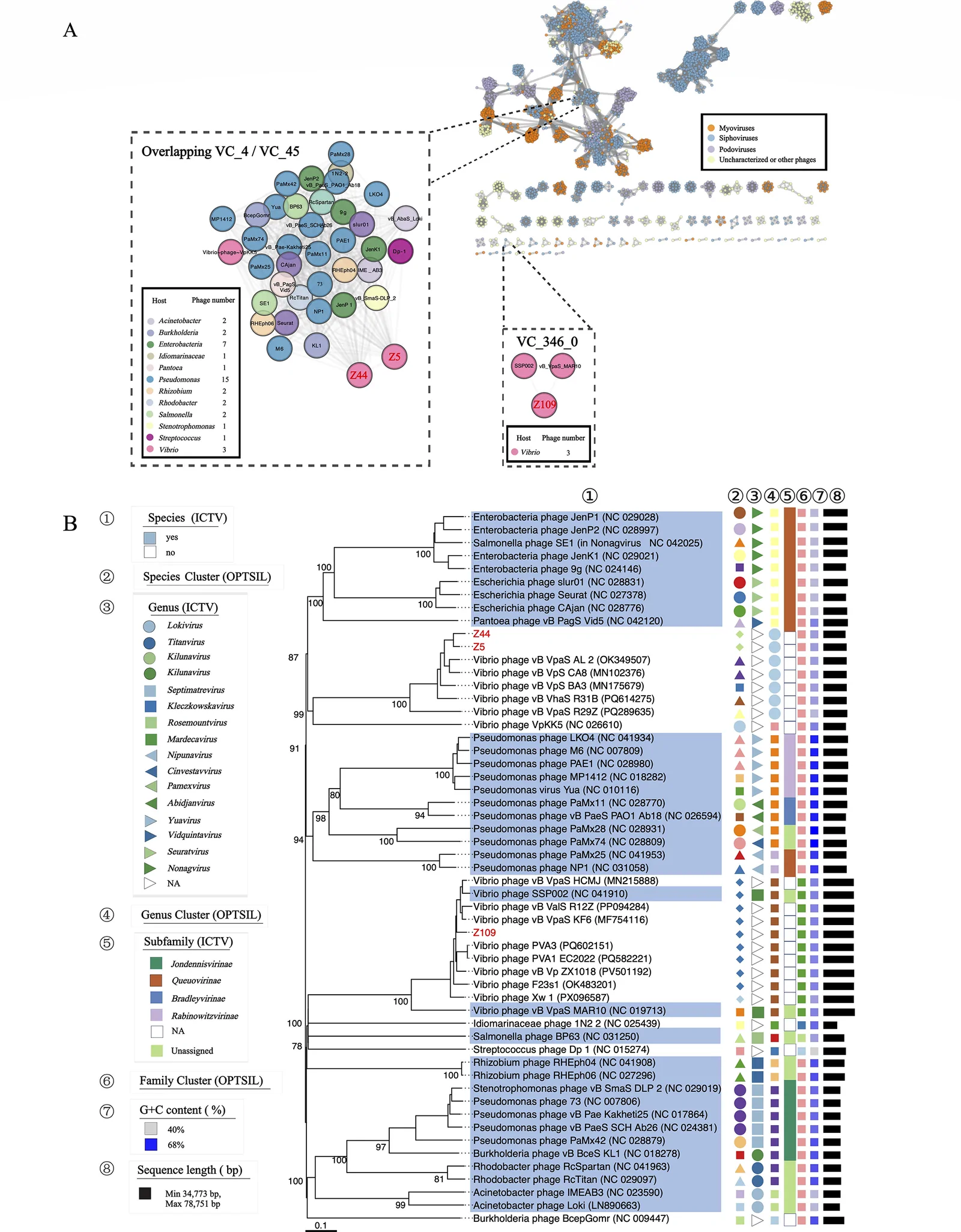
Protein-sharing network and whole-genome phylogeny of phages Z5, Z44, and Z109. (**A**) Protein-sharing network of phage genomes generated using vConTACT2. Each node represents a phage genome, and edges indicate significant similarity inferred from shared protein clusters. Phages Z5 and Z44 cluster with 37 related phages within overlapping viral clusters VC_4 and VC_45, whereas Z109 forms a separate cluster (VC_345_0) together with two related phages. Enlarged views show subnetworks containing each focal phage. (**B**) Whole-genome phylogeny inferred using the GBDP approach implemented in VICTOR based on amino acid sequences. ICTV taxonomic assignments, OPTSIL clustering results, as well as GC content and genome sizes are indicated. “Unassigned” indicates phages not yet assigned to a defined genus according to the ICTV, whereas “NA” indicates viruses not currently recognized as taxa by ICTV.

To determine the phylogenetic placement of Z5, Z44, and Z109, a protein-sharing network was constructed using vConTACT2 based on shared protein clusters, including the three genomes of Z5, Z44, Z109, and 2,616 reference phages (Fig. 4A; Table S3). Network analysis revealed that Z5 and Z44 were grouped together within overlapping viral clusters (VCs), specifically Vc_4 and Vc_45 along with 37 related phages, including *Vibrio* phage VpKK5. In contrast, Z109 formed a distinct cluster (Vc_345_0) with two related phages.

**Fig 5:**
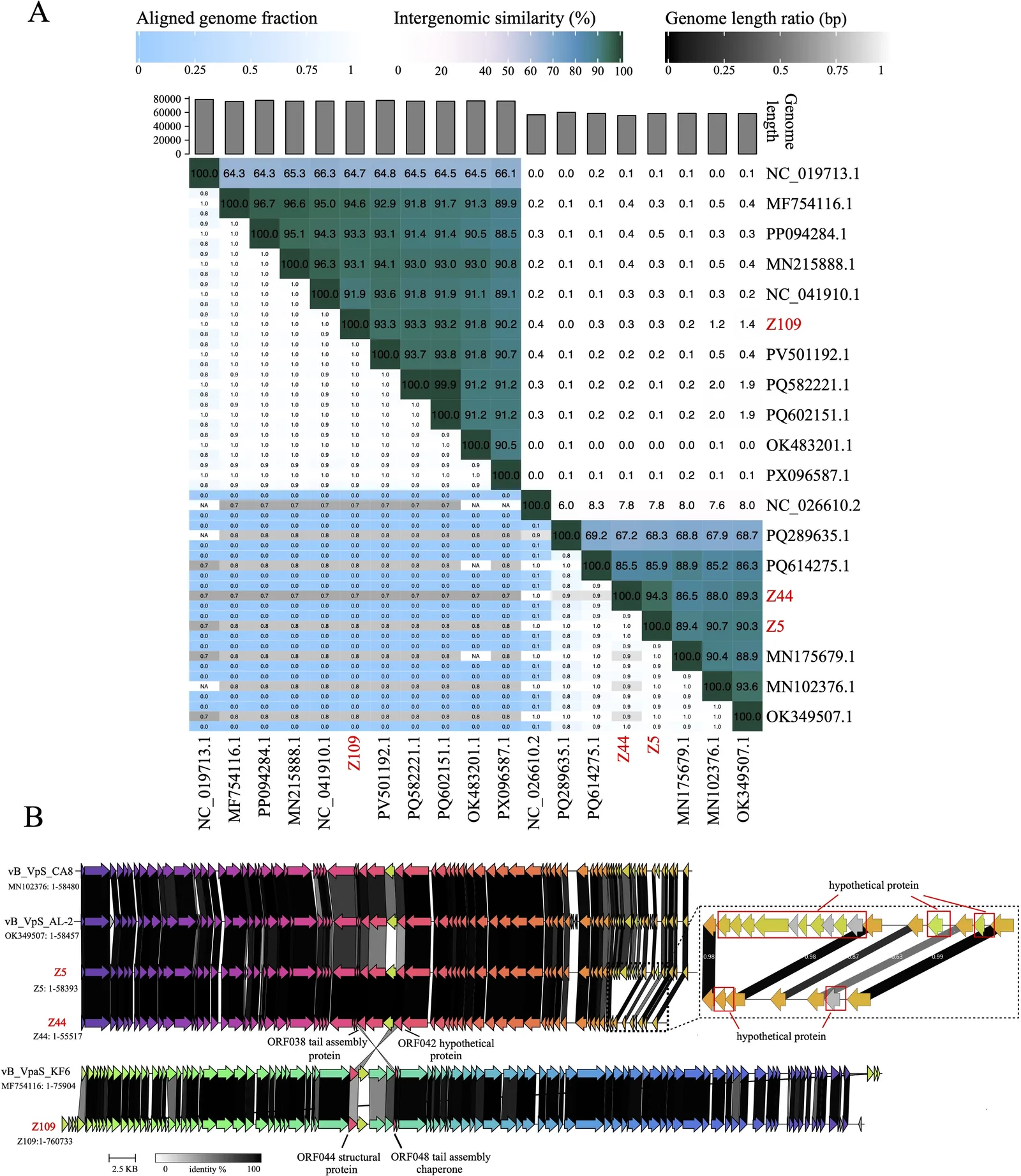
Intergenomic comparison of phages Z5, Z44, and Z109 with related phages. (**A**) Intergenomic similarity among phages Z5, Z44, Z109, and closely related phages. Heatmap of pairwise intergenomic similarities calculated using the VIRIDIC web service. (**B**) Comparative genome alignment of phages Z5, Z44, and Z109 with related phages using Clinker. The dashed box indicates an enlarged region highlighting structural differences between phages Z5 and Z44. ORFs with high sequence similarity are shown as arrows of the same color, with shading intensity reflecting the degree of sequence similarity.

To further investigate the evolutionary relationships of the three phages, a genome-based phylogenetic tree was constructed using the GBDP approach, incorporating representative genomes from 39 viral clusters and 12 additional *Vibrio* phages retrieved from GenBank. OPTSIL clustering based on GBDP distances generated 39 species-level clusters and 12 genus-level clusters (Fig. 4B). Within this framework, Z5 and Z44 clustered with *Vibrio* phages VpKK5, AL2, CA3, BA3, R31B, and R21Z (45–47), whereas Z109 clustered with phages HCMJ, SSP002, R12Z, KF6, PVA3, PVA1, ZX1018, F23s1, and Xw1 (48–50). According to the ICTV criteria for tailed bacteriophage taxonomic classification (36), phages sharing ≥70% intergenomic similarity belong to the same genus, whereas those sharing ≥95% similarity belong to the same species. VIRIDIC analysis showed that Z5 and Z44 share their highest intergenomic similarities with phages CA8 (90.8%) and AL2 (90.3%), respectively (Fig. 5A). Based on ICTV thresholds, both Z5 and Z44 were identified as novel species within the subfamily *Queuovirinae*. In contrast, Z109 exhibited high similarity to phage KF6 (94.6%), indicating that Z109 belongs to the genus *Mardecavirus*.

### Phage *in vitro* bactericidal activity

**Fig 6:**
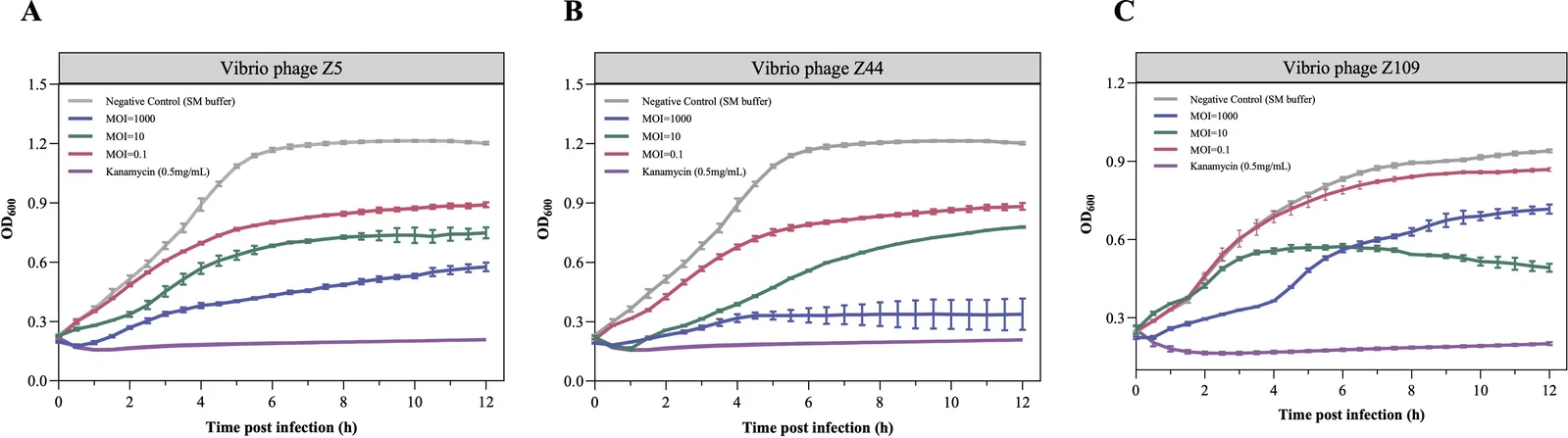
*In vitro* bactericidal effects of phages Z5, Z44, and Z109 against their respective *Vibrio parahaemolyticus* hosts at different MOIs. (**A–C**) Killing curves showing the effects of phages Z5 (**A**), Z44 (**B**), and Z109 (**C**) on the growth of the respective *V. parahaemolyticus* hosts at MOIs of 0.1, 10, and 1,000 over a 12 h incubation period. Bacterial growth was monitored by measuring OD₆₀₀ at 0.5 h intervals.

**Fig 7:**
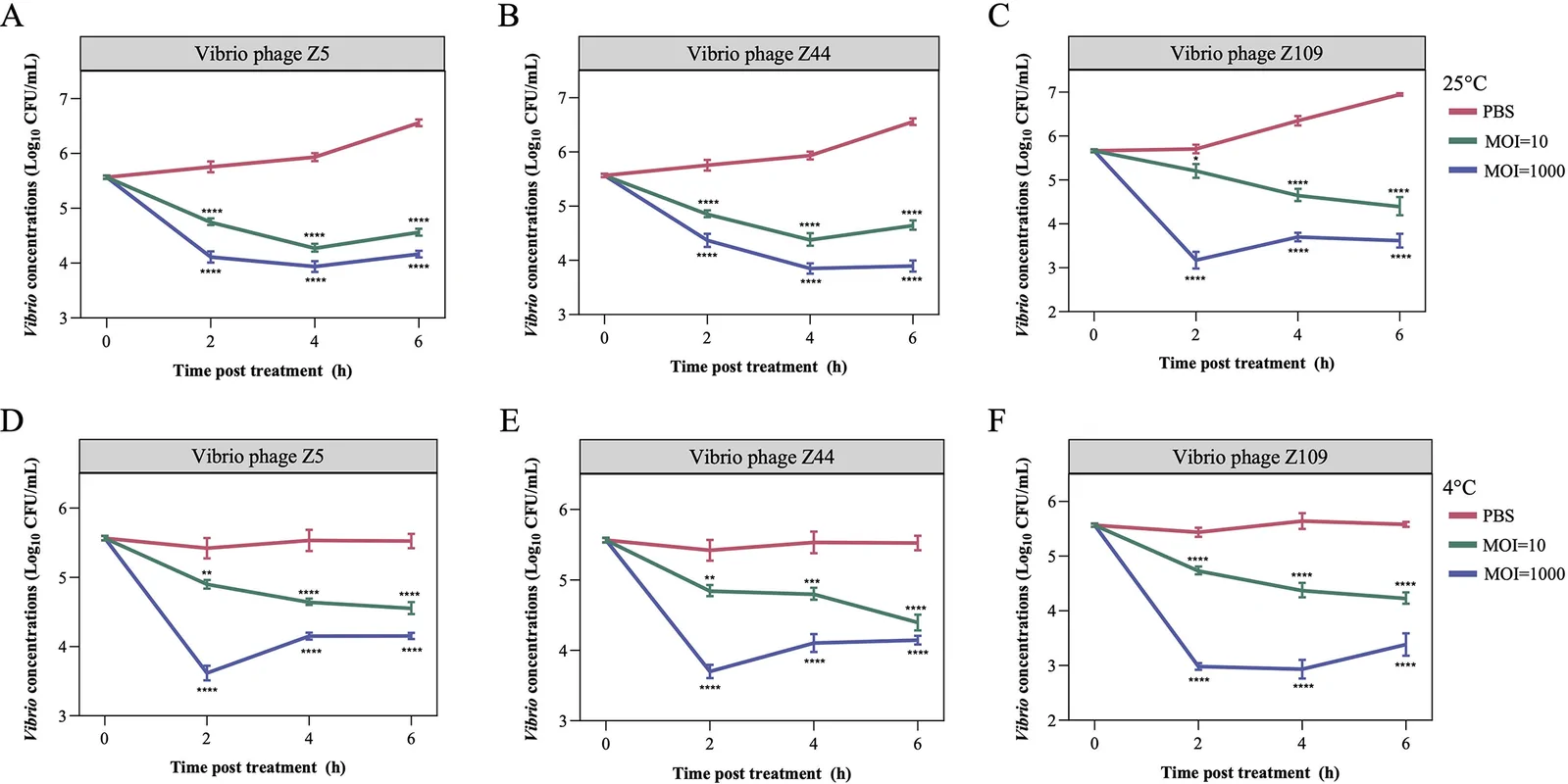
Inhibition of *Vibrio parahaemolyticus* contamination on raw salmon surfaces by phages Z5, Z44, and Z109. (**A–C**) Inhibition of *V. parahaemolyticus* on raw salmon surfaces by phages Z5, Z44, and Z109 at 25°C under different MOIs. (**D–F**) Inhibition at 4°C of *V. parahaemolyticus* on raw salmon surfaces by phages Z5, Z44, and Z109 under different MOIs. Statistical significance was evaluated by one-way ANOVA, followed by Dunnett’s post hoc test, with each phage-treated group compared to the PBS control at the corresponding time point. Data are presented as mean ± SD (*n* = 3). Significance levels are indicated as follows: ns, not significant; *P < 0.05; **P < 0.01; ***P < 0.001; ****P < 0.0001.

The *in vitro* bactericidal activities of the three phages against *V. parahaemolyticus* were evaluated at different MOIs using killing curve analysis (Fig. 7). During the initial phase (0–6 h post-phage addition), all three phages exhibited dose-dependent inhibition of bacterial growth, with higher MOIs associated with greater suppression of host proliferation. At an MOI of 1,000, phages Z5 and Z44 induced rapid and sustained lysis of *V. parahaemolyticus*, maintaining OD₆₀₀ values below 0.6 and 0.4, respectively, throughout the 12 h treatment period (Fig. 6A and B). Notably, even at an MOI of 0.1, both phages retained measurable bactericidal activity, resulting in an approximately 31% reduction in OD₆₀₀ at 12 h compared with the negative control.

In contrast, phage Z109 exhibited distinct killing kinetics. Although treatment at an MOI of 1,000 led to stronger growth inhibition than an MOI of 10 during the first 6 h (OD₆₀₀ < 0.6), bacterial regrowth occurred at later time points. Consequently, OD₆₀₀ values in the MOI 1,000 group ultimately exceeded those in the MOI 10 group (Fig. 6C). This non-monotonic dose-response relationship indicates a more complex phage-host interaction for Z109 at a high phage dose (MOI = 1,000).

### Bactericidal efficacy of three phages in raw salmon model

The bactericidal efficacy of phages Z5, Z44, and Z109 against *V. parahaemolyticus* on raw salmon surfaces was evaluated at 25°C and 4°C, representing room-temperature handling and refrigerated storage conditions, respectively (Fig. 7).

At 25°C, bacterial concentrations in the PBS control group increased progressively, rising by more than one order of magnitude to approximately 6.5 log₁₀ CFU/mL within 6 h, indicative of rapid proliferation of *V. parahaemolyticus* on the salmon surface. In contrast, phage-treated groups demonstrated significant, dose-dependent reductions in bacterial load (*P* < 0.05). At an MOI of 1,000, bacterial counts in the Z5-, Z44-, and Z109-treated groups decreased by 1.52, 1.39, and 2.53 log₁₀ CFU/mL, respectively, within 2 h of treatment compared with the corresponding PBS control (*P* < 0.0001), corresponding to reductions of approximately 97.0%, 95.9%, and 99.7%, respectively. Compared to the PBS control, reductions reached 2.39, 2.66, and 3.33 log₁₀ CFU/mL at 6 h post-treatment, respectively (*P* < 0.0001), equivalent to reductions of approximately 99.6%, 99.8%, and 99.95%, respectively. At a lower MOI of 10, all three phages continued to exhibit significant bactericidal activity (*P* < 0.05), although reductions were less pronounced than those observed at MOI 1,000. At 6 h, bacterial concentrations in the Z5-, Z44-, and Z109-treated groups were 4.56, 4.64, and 4.39 log₁₀ CFU/mL, corresponding to reductions of 1.99, 1.91, and 2.55 log₁₀ CFU/mL compared with the control (*P* < 0.0001), respectively, equivalent to approximately 99.0%, 98.8%, and 99.7%, respectively.

At 4°C, bacterial concentrations in the PBS control group remained stable over the experimental period, consistent with limited growth under refrigeration. Nevertheless, phage application resulted in significant reductions in bacterial load on raw salmon surfaces (*P* < 0.01). At an MOI of 1,000, bacterial loads in Z5-, Z44-, and Z109-treated groups were reduced by 1.37, 1.38, and 2.19 log₁₀ CFU/mL, respectively, at 6 h post-treatment compared with the PBS control (*P* < 0.0001), corresponding to reductions of approximately 95.7%, 95.8%, and 99.4%, respectively. At an MOI of 10, reductions of 0.97, 1.13, and 1.35 log₁₀ CFU/mL were observed at 6 h post-treatment compared with the PBS control at the corresponding time point (*P* < 0.0001), equivalent to reductions of approximately 89.3%, 92.6%, and 95.5%, respectively.

Collectively, these findings indicate that, under conditions representative of seafood handling and cold-chain storage, all three phages maintain substantial antibacterial activity in the salmon matrix across the tested temperature range, highlighting the potential application of phage-based interventions as practical biocontrol strategies to reduce contamination in seafood.

### Prevention and disruption of biofilm by phages

**Fig 8:**
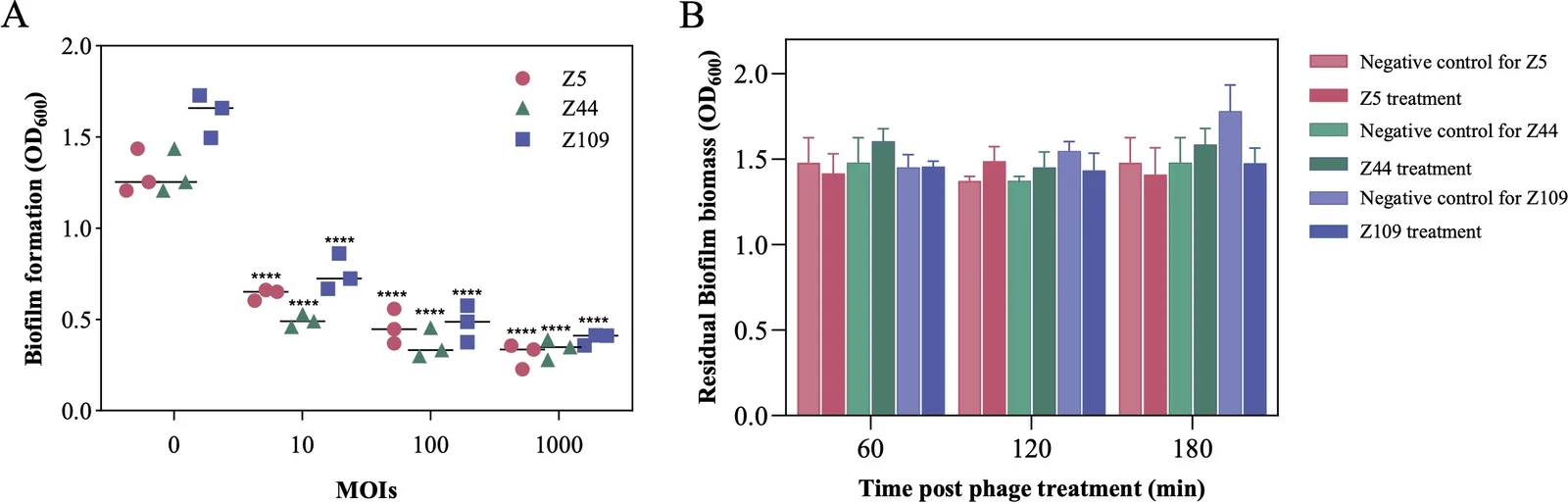
Prevention (**A**) and disruption (**B**) of *Vibrio parahaemolyticus* biofilms by phages Z5, Z44, and Z109. (**A**) Effects of phages at different MOIs on biofilm formation. (**B**) Residual biofilm biomass of pre-formed biofilms at 60, 120, and 180 min post-phage treatment. Statistical analyses were performed using one-way ANOVA, followed by Dunnett’s post hoc test for panel A and two-way ANOVA, followed by Tukey’s multiple comparisons test for panel B. Data are presented as mean ± standard deviation (SD) from three biological replicates (*n* = 3). Significance levels are indicated as follows: ns, not significant; *P < 0.05; **P < 0.01; ***P < 0.001; ****P < 0.0001.

The effects of phages Z5, Z44, and Z109 on *V. parahaemolyticus* biofilms were evaluated by assessing both biofilm prevention and biofilm disruption. As shown in Fig. 8A, all three phages significantly inhibited biofilm formation in a dose-dependent manner, with OD_600_ values in the phage-treated groups markedly lower than those in the PBS control (*P* < 0.001). Increasing the MOI further enhanced the inhibition of biofilm formation, indicating that early phage application effectively interferes with initial biofilm establishment.

In contrast, phage treatment of pre-formed biofilms resulted in limited reductions in biofilm biomass (Fig. 8B), and no clear dose-dependent trend was observed under the tested conditions. This observation is consistent with previous studies reporting reduced susceptibility of mature biofilms to phage treatment (51). Collectively, these findings indicate that although the three phages are highly effective in preventing biofilm formation, their capacity to disrupt established biofilms is comparatively limited, underscoring the importance of early phage intervention for controlling *V. parahaemolyticus* biofilms in food-related environments.

## DISCUSSION

*V. parahaemolyticus* is a leading cause of seafood-associated foodborne outbreaks worldwide and remains a persistent challenge to seafood safety. Epidemiological studies have reported its frequent detection in various food products and its association with gastroenteritis outbreaks in China (52–54). The increasing prevalence of antibiotic-resistant *V. parahaemolyticus* strains (55, 56) underscores the need for alternative biocontrol strategies that ensure food safety without adversely affecting the sensory or nutritional properties of seafood products. Bacteriophages, as natural predators of bacteria with high host specificity and inherent safety for humans and animals, have therefore attracted increasing attention as promising alternatives to conventional food-grade antimicrobials in aquatic food systems (57).

In the present study, three novel *Vibrio* phages, Z5, Z44, and Z109, were isolated from wastewater collected at seafood markets using *V. parahaemolyticus* as the host strain and were subsequently characterized. According to the current ICTV taxonomy, Z5 and Z44 represent novel species within the subfamily *Queuovirinae*, whereas Z109 is assigned to the genus *Mardecavirus*. This taxonomic diversity provides a foundation for the future development of phage cocktails with enhanced bactericidal efficacy (57). For food-related applications, phages should not harbor antibiotic resistance genes or virulence factors (58, 59). Genome analysis revealed no detectable antibiotic resistance or virulence-associated genes in the phage genomes, suggesting their genomic safety.

Because phages used in practical settings may encounter variable environmental conditions, comprehensive characterization of their biological properties is essential (60). All three phages retained high activity across a broad temperature range (4°C–60°C) and pH range (4–10), supporting their suitability for applications spanning from marine aquaculture (10°C–25°C, pH 6.5–7.4) (61) and food storage and handling conditions (4°C–25°C). Compared with previously reported *V. parahaemolyticus* phages, including vB_VpaM_PVP-XSN, OMN, and PG288, which remain stable up to 50°C (62–64), the three phages characterized here exhibited greater thermal tolerance, maintaining activity at 60°C. Previous studies have indicated that phages with large burst sizes and short latent periods generally exhibit strong lytic activity and superior bactericidal efficacy (65). Consistent with this, phages Z5 and Z44, which exhibited short latent periods (~10 min) and high burst sizes (267 ± 15 and 688 ± 50 PFU per infected cell, respectively) produced substantial inhibition of *V. parahaemolyticus* growth, resulting in approximately 52.1% and 71.9% reductions in bacterial growth within 12 h at an MOI of 1,000, respectively, as demonstrated by OD_600_ measurements. These characteristics support the strong lytic potential of Z5 and Z44 and their potential utility as effective biocontrol agents against *V. parahaemolyticus* in food-related applications.

In practice, phage treatment does not invariably result in complete elimination of target bacteria, reflecting the co-evolutionary dynamics between bacteria and their phages. One widespread phage counter-defense strategy (66–71) involves genomic DNA modification. Recent studies have shown that certain phages incorporate modified nucleotides, such as 2′-deoxy-7-deazaguanine (dG^+^) and its derivatives (including dADG, dPreQ₀, and dPreQ₁), in place of canonical guanine, thereby conferring resistance to diverse bacterial restriction endonucleases (72). Genome analysis of phages Z5 and Z44 identified multiple genes associated with 7-deazaguanine DNA modification pathways, including an organometallic radical-activating enzyme, a QueC-like protein, 6-pyruvoyl tetrahydropterin synthase, GTP cyclohydrolase I type 1, and a putative tRNA ribosyltransferase. The co-occurrence of these genes suggests that Z5 and Z44 may encode a functional 7-deazaguanine DNA modification system. Consistent with this observation, vConTACT2 clustering grouped Z5 and Z44 within the same viral cluster as phage *Enterobacteria* phage 9g (NC_024146), the first reported phage to encode such a system conferring resistance to host restriction mechanisms (72). This DNA modification system in Z5 and Z44 may be associated with host restriction-modification systems and potentially contribute to genome stability during infection, thereby contributing to their observed lytic performance under application-relevant conditions in aquaculture and food supply chains.

Notably, in contrast to Z5 and Z44, phage Z109 exhibited a distinct dose-dependent bactericidal pattern. At an MOI of 1,000, pronounced early bactericidal activity was observed during the early stages of infection. However, this effect declined markedly at later time points. In contrast, although an MOI of 10 produced weaker initial inhibition, the late-stage suppressive effect was markedly stronger than that observed at an MOI of 1,000. This phenomenon, characterized by strong early bactericidal activity at high doses, followed by rapid attenuation, may be associated with host-encoded antiphage defense mechanisms, including those derived from prophage elements within the host genome.

Genome analysis revealed that the host strain *Vibrio parahaemolyticus* vp-HL-202005 harbors at least four putative prophage regions (Fig. S1), collectively encoding genes previously implicated in antiphage defense. These include Cro/CI family transcriptional regulators associated with superinfection exclusion (73), the abortive infection protein AbiV (74), which is capable of triggering programmed cell death during the early stages of phage DNA replication, and a type II toxin-antitoxin system (RelE/ParE–Phd/YefM) (75), which can inhibit cellular translation or induce host suicide following phage infection. Furthermore, characterization of receptor-binding proteins and their corresponding host receptors will be essential to strengthen the mechanistic understanding of phage-host interactions. Together, these prophage-encoded elements may contribute to the observed differences in infection dynamics under different MOI conditions.

Therefore, it can be hypothesized that at high phage doses (MOI 1,000), strong infection pressure promotes activation of abortive infection systems (e.g., AbiV) and toxin-antitoxin modules (RelE/ParE–Phd/YefM). Such responses may drive infected cells into dormancy or programmed cell death, thereby limiting productive phage replication and reducing bactericidal efficacy at later stages of infection. In contrast, under lower infection pressure (MOI 10), host antiphage defenses may be less strongly induced, allowing more sustained phage replication during the late phase and consequently prolonged bactericidal activity. Taken together, these findings suggest that prophage-derived antiphage factors, including S24, Cro/CI, AbiV, and RelE/ParE–Phd/YefM systems, may contribute to diminished late-stage bactericidal performance under high MOI conditions. These observations highlight the importance of rational phage dose optimization to improve the stability and effectiveness of phage-based biocontrol strategies. However, further functional studies are required to confirm the underlying molecular mechanisms.

*V. parahaemolyticus* readily forms persistent biofilms on both biotic and abiotic surfaces, posing a significant challenge to seafood safety and sanitation. Effective control of biofilm-associated contamination on seafood products and food-contact surfaces is therefore essential to reduce foodborne risk and spoilage-related losses (76–79). In the present study, all three phages demonstrated substantial application potential in food-related contexts. In the raw salmon model, phage treatment significantly reduced the abundance of *V. parahaemolyticus*, indicating effective suppression of bacterial proliferation on seafood surfaces. These findings highlight the feasibility of phage-based biocontrol as a complementary strategy to conventional cold-chain hygiene measures.

Beyond their direct bactericidal activity against planktonic *V. parahaemolyticus*, the phages exhibited strong antibiofilm activity during biofilm formation. Given the persistence of *V. parahaemolyticus* biofilms on seafood-processing equipment and food-contact surfaces, this characteristic is particularly important. By interfering with early bacterial attachment and markedly reducing biofilm biomass, these phages provide an additional protective measure applicable to seafood safety management. Overall, the pronounced bactericidal activity in the raw salmon model and the strong anti-biofilm effects suggest considerable potential for application within the seafood supply chain.

### Conclusion

In the present study, three novel lytic phages, Z5, Z44, and Z109, targeting *V. parahaemolyticus* were isolated and comprehensively characterized. Their short latent periods, large burst sizes, strong lytic capacity, and favorable genomic profiles underscore their potential as candidates for phage-based biocontrol applications. All three phages effectively reduced *V. parahaemolyticus* contamination on salmon surfaces at 25°C and 4°C and significantly inhibited biofilm formation, demonstrating practical efficacy in food matrices. These findings provide a feasible alternative to conventional decontamination approaches, enabling the effective control of *V. parahaemolyticus* contamination while preserving food quality. Future studies should focus on the development and optimization of phage cocktails incorporating Z5, Z44, and Z109 to enhance their applicability in the food industry.

## Data Availability

The complete genome sequences of phages Z5, Z44, and Z109 were deposited in the GenBank database under accession numbers PX979761, PX979762, and PX979763, respectively.

## SUPPLEMENTAL MATERIAL

Figure S1 spectrum.02617-26-s0001.docx Putative prophage-encoded antiphage defense genes in the genome of Vibrio parahaemolyticus vp-HL-202005.

Supplemental material spectrum.02617-26-s0002.xlsx Tables S1 to S3, genome annotations of phages Z5, Z44, and Z109, and prophage genome annotations.

ASM does not own the copyrights to Supplemental Material that may be linked to, or accessed through, an article. The authors have granted ASM a non-exclusive, world-wide license to publish the Supplemental Material files. Please contact the corresponding author directly for reuse.
