# Supplementary material for: Genomic and biological characterization of three lytic phages targeting Vibrio parahaemolyticus and their application in a raw salmon model
Source: Microbiol Spectr. 2026 Sep 22;14(11):e02617-26. doi: 10.1128/spectrum.02617-26 (PMC13729227; doi:10.1128/spectrum.02617-26)
Supplement: Supplementary file 1 [file spectrum.02617-26-s0001.docx]

**Figure S1. Putative prophage-encoded antiphage defense genes in the genome of *Vibrio parahaemolyticus* vp-HL-202005.** The genomic organization of four putative prophage regions is shown. Arrows indicate predicted open reading frames (ORFs) and their transcriptional orientation. Prophage 1 encodes a superinfection exclusion (SIE) module, including a Cro/CI family transcriptional regulator and the Cor protein. Prophage 3 harbors an AbiV family abortive infection protein and a type II toxin–antitoxin system (RelE/ParE–Phd/YefM). The remaining prophage regions primarily encode structural or replication-related proteins and are shown for genomic context.
